# Evaluating the performance of the Bayesian mixing tool MixSIAR with fatty acid data for quantitative estimation of diet

**DOI:** 10.1038/s41598-020-77396-1

**Published:** 2020-11-27

**Authors:** Alicia I. Guerrero, Tracey L. Rogers

**Affiliations:** 1 Centro de Investigación y Gestión de Recursos Naturales (CIGREN), Instituto de Biología, Facultad de Ciencias, Universidad de Valparaíso, Gran Bretaña, 1111, Playa Ancha, Valparaíso, Chile; 2 Evolution and Ecology Research Centre, School of Biological, Earth and Environmental Sciences, University of New South Wales, Sydney, 2052 Australia

**Keywords:** Ecosystem ecology, Tropical ecology, Stable isotope analysis

## Abstract

We test the performance of the Bayesian mixing model, MixSIAR, to quantitatively predict diets of consumers based on their fatty acids (FAs). The known diets of six species, undergoing controlled-feeding experiments, were compared with dietary predictions modelled from their FAs. Test subjects included fish, birds and mammals, and represent consumers with disparate FA compositions. We show that MixSIAR with FA data accurately identifies a consumer’s diet, the contribution of major prey items, when they change their diet (diet switching) and can detect an absent prey. Results were impacted if the consumer had a low-fat diet due to physiological constraints. Incorporating prior information on the potential prey species into the model improves model performance. Dietary predictions were reasonable even when using trophic modification values (calibration coefficients, CCs) derived from different prey. Models performed well when using CCs derived from consumers fed a varied diet or when using CC values averaged across diets. We demonstrate that MixSIAR with FAs is a powerful approach to correctly estimate diet, in particular if used to complement other methods.

## Introduction

Quantitative dietary studies are important for ecosystem-based management and are needed to predict potential impacts on predator–prey dynamics^1,2^. However, accurate dietary estimations are difficult to obtain as most of the traditional methods available, such as scat and stomach content analyses, provide a qualitative identification of the most recent dietary intake^3,4^ or provide quantitative information that is potentially biased towards prey types containing hard parts^3, 5^. Thus, biochemical methods have been proposed as a solution to some of these constraints. Biochemical compounds, such as fatty acids (FAs) and stable isotopes, are effective dietary tracers because they are predictably modified when transferred from the prey (“source”) to the tissues of the predator (“consumer”)^6,7^.

Stable isotope analysis has been widely used as a quantitative approach for ecological studies^7^; mainly through the development of robust analytical tools such as the linear mixing model IsoSource^8^, or the Bayesian mixing models SIAR^9^ and MixSIR^10^. More recently, Stock et al.^11^ developed another Bayesian framework, MixSIAR, which integrates a set of parameterizations that improve on the error structure of its predecessors SIAR and MixSIR, in terms of their assumptions about the predation process^12^. This new generation of Bayesian tracer mixing model has already been widely applied to stable isotope data for ecological studies^13–15^, however, although it can be used with other biochemical tracers, little is known about its performance with FA data.

Unlike stable isotopes, FAs have been used mostly as a qualitative tool, where similarities in FA signatures between sources and consumers are evaluated through the application of multivariate analyses e.g. Refs.^16–19^. Although FAs have proven to be a useful tool to elucidate dietary patterns^20^, their quantitative use in foraging ecology studies has been slow compared to stable isotope studies. The first quantitative framework developed for FA data was the “Quantitative Fatty Acid Signature Analysis (QFASA)” approach^4^, which uses a multivariate least-squares model to estimate the contribution of sources to the consumer’s FA signature^21^. Bayesian mixing models, although originally developed for stable isotope data, are being increasingly applied to FA data to estimate the diet of consumers^22–25^. Galloway et al.^22^ quantified the diet composition of herbivorous zooplankton using “Fatty Acid Source Tracking Algorithm in R” (FASTAR). FASTAR uses the stable isotope equations of MixSIR or SIAR to calculate the proportional contribution of a consumer’s potential food sources^10^. However, the performance of FASTAR, using the MixSIR model and FA data, was less accurate than QFASA at predicting the diet of captive belugas^26^. Neubauer and Jensen^25^ developed the mixing model framework “fastinR” that integrates stable isotope and FA data into a joint model to estimate diet. This model accounts for compositional constraints on FA data using an additive log-ratio transformation which makes the data approximately normal. It is unclear how dietary estimations could be affected by data transformation, as this tends to give artificial weight to FAs in small quantities, hence, minimizing the importance of more abundant FAs^27^. FastinR also incorporates a multiplicative constant to account for trophic modification (differences in tracer values between consumer and sources^28^) of FAs, which differs from models developed for stable isotope data. This model, however, has gathered little attention^29^ and, to our knowledge, has not been tested in further studies.

For stable isotopes, the tracer value of the consumer equals the tracer value of the prey plus trophic modification (also named trophic fractionation, trophic discrimination, or discrimination factors). Thus, the tracer values of the consumer follow Eq. (1):

1 $$y_{j} = \mathop \sum \limits_{k} p_{k} \left( {\mu_{jk} + t_{j} } \right)$$

where $y_{j}$ is the tracer value *j* of the consumer *y*; $p_{k}$ is the proportional contribution of the source *k* to the diet of the consumer; $\mu_{jk}$ is the source *k* mean for tracer *j*; and $t_{j}$ is the trophic modification value for tracer *j*.

**Figure 1:**
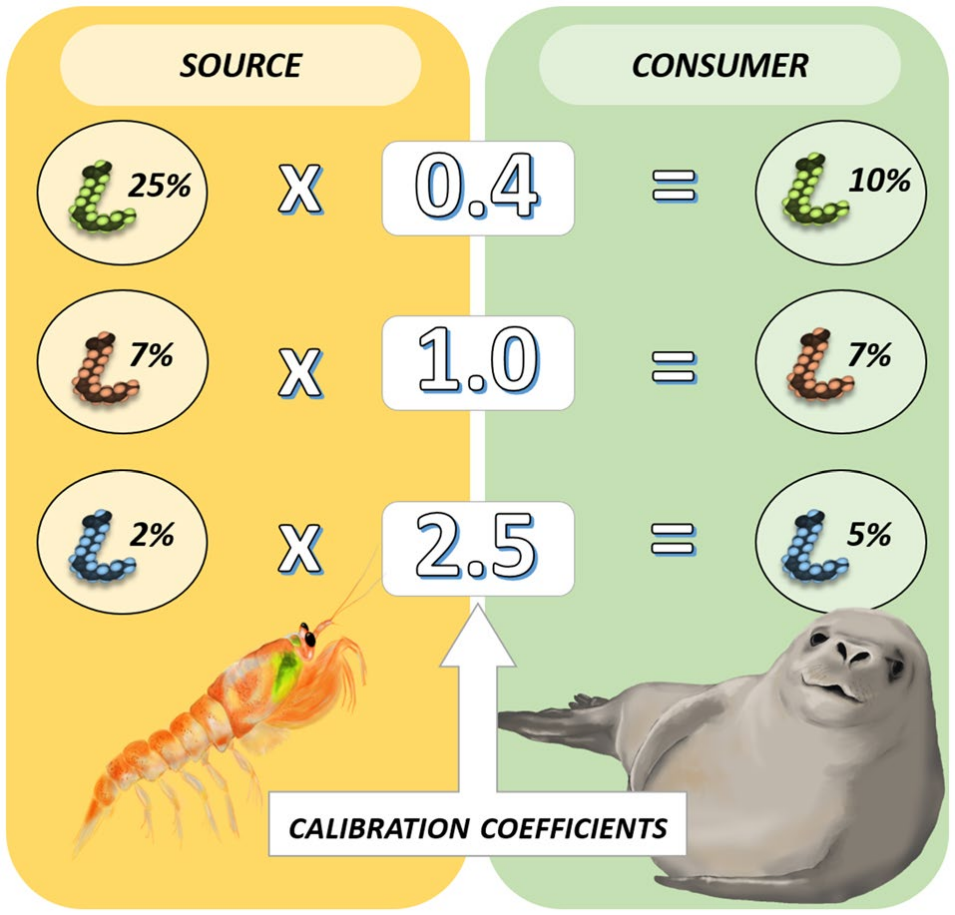
Diagram of trophic modification of fatty acids (FAs) when transferred from prey (source) to predator (consumer). FAs can be lower, equal or higher in the consumer compared to sources, depending on the consumer’s metabolism and nutritional state. Calibration coefficients (CCs) are a simple mathematical calculation to represent the modification of each FA from prey to predator, and they are calculated by means of feeding trials where consumers are fed certain diet until FA turnover is considered complete. The case in the figure applies for a consumer fed 100% a solely prey. Images by Alicia Guerrero.

FAs, however, are predominantly presented as proportional values, and depending on the consumer’s metabolism, FAs may be higher or lower than the proportions found in the prey. In the FA literature, the term used for trophic modification is calibration coefficient (CC)^4^. CCs are multiplicative values, where the tracer value of the consumer equals the tracer value of the prey multiplied by its corresponding CC (Fig. 1), as in Eq. (2):

2 $$y_{j} = \mathop \sum \limits_{k} p_{k} \left( {\mu_{jk} \times t_{j} } \right)$$

Therefore, when using mixing models, most scientists have opted for using a FA resource library where trophic modification is already integrated (i.e. consumers fed single prey diets are used as sources, thus trophic modification is zero) e.g. Ref.^23,30^ or have simply omitted the use of CCs e.g. Ref.^31^.

To date, the most popular and well evaluated model to estimate diet using FAs is the numerical-optimization based method QFASA^30^. The QFASA approach has limitations associated with not being able to account for multiple sources of uncertainty, as well as the inability to incorporate ecological mechanisms into the models^25^. Given the complexity of biological systems, particularly that they are often influenced by multiple factors, it would be advantageous to use modelling approaches that allow for the inclusion of ecological information. MixSIAR models diet proportions as a function of ecologically meaningful factors^11^; previous knowledge can be incorporated into models as informative priors, as can the uncertainty in source values, unlike QFASA which can use only mean source values^30^. Despite the promise of MixSIAR with FA data, it has not been widely implemented, hence, its performance is still an open question.

Testing the performance of diet estimation methods is an important step prior to their implementation with field data. Here we aim to assess how incorporating the Bayesian mixing model approach, MixSIAR, performs in estimating diet based on FA data, by comparing model-derived dietary predictions with known diet. While to date most diet-estimation methods have been applied to single species or taxonomic groups e.g. Ref.^23,25,26,29,32,33^, we estimate dietary compositions across taxa including fish, birds and mammals. To evaluate MixSIAR’s performance in predicting diet with FAs, we use published data from six species undergoing feeding experiments. We focus here on some aspects that could influence model prediction, including the effect of: (a) using CCs derived from a monotypic or a mixed diet; (b) modelling diet when FA turnover is incomplete; (c) consuming a low-fat diet; (d) inclusion of prior information in the model; and (e) other physiological and methodological factors that can affect the interpretation of diet estimates.

## Methods

### Data preparation

Using published literature, we selected six feeding trial studies, where animals were fed single or multiple prey types, and the FA signature of the consumer’s fat was analysed at some point of the experiment. In the published literature, FA signatures are usually presented as mean and standard deviations. Since MixSIAR requires consumer values to be input as raw data, we generated normally-distributed random values for each FA, using the ‘rnorm’ function in R^34^, based on the mean, standard deviation and sample size provided in each published study. Thus, simulated data follow MixSIAR assumptions about independence and normality; however, actual FA profiles do not. Therefore, our simulated data could act differently to compositional FA data. We assessed the effect of using simulated data derived from means and standard deviations instead of actual FA profiles, using experimental data from Stowasser et al.^35^ and the diet estimations obtained from both sets of data did not differ significantly (See details in Supplementary material 1). Thus, we assume that if actual FA profiles were used instead of simulated data, the outcomes would be the same or similar.

To quantify the relative contribution of sources to a consumer or “mixture”^10,28^, mixing models require tracer data (here, FA signature) of consumers, sources, and values for trophic modification (here, CCs). All CCs were obtained from published studies, where animals were fed a single or mixed diet over a period of time considered sufficient to represent complete FA turnover (See each case for details). Since MixSIAR treats fractionation as additive values, and CCs are multiplicative, we took sources to predator space by multiplying source FA values by its corresponding CC and thus used the resulting values as ‘sources’. Thus, since our sources already accounted for trophic modification, we set modification values to zero in all our models.

For each analysis, we used only those FAs present in amounts higher than 0.5% in the consumer, and those classified as “dietary” and “extended dietary”, according to Iverson et al.^4^. “Dietary” FAs are those that consumers are unable to synthesise whereas “extended dietary” FAs are both dietary and endogenous in origin^4^. Additionally, we excluded FAs whose CC was higher than 2 units, since this indicates that the consumer has at least twice the proportion of that FA compared to its prey, suggesting that the origin is either endogenous (rather than dietary) or the consumer is exhibiting preferential accrual.

Mixing models require sources to be significantly different^36^. Therefore, we assessed the differences in FA composition among sources using Permutational Multivariate Analysis of Variances (PERMANOVA) with the *adonis* function of the R package “vegan”^37^. When the number of sources was higher than two, we conducted pairwise multilevel comparisons in order to identify which prey species were different. These comparisons were conducted applying corrections for multiple testing, with the R package “pairwiseAdonis”^38^.

### Diet estimation

To analyse the contribution of sources to the diet of consumers for all our cases, we used the Bayesian mixing model MixSIAR GUI v3.1^28^. FA values of consumers were input as raw data. Although FA data are correlated, MixSIAR assumes multivariate normality, which accounts for the fact that tracer values can co-vary. Sources were input as mean and standard deviation, as suggested by Stock et al.^11^ when using proportional data, thus, FAs are assumed to be independent. We used non-informative priors, unless otherwise noted. The model is fit via Markov Chain Monte Carlo, which estimates entire posterior distributions for each variable, given the data. We set error structure as a multiplicative process (‘Residual*Process’), as this is an ecologically realistic scenario^12^. Model convergence was assessed via Gelman–Rubin and Geweke diagnostics^39,40^. For a more comprehensive understanding, we explain each feeding experiment in the results section, as well as further details about model settings. Datasets including FAs of consumers and sources (with incorporated CCs) are available as Supplementary Material 2. Data analyses were conducted using JAGS and R software^34,41^. The posterior distribution medians and ranges obtained from each case are available as Supplementary material 3.

## Results

### Case 1: spectacled eiders, from Wang et al***.***^42^

#### The experiment

**Figure 2:**
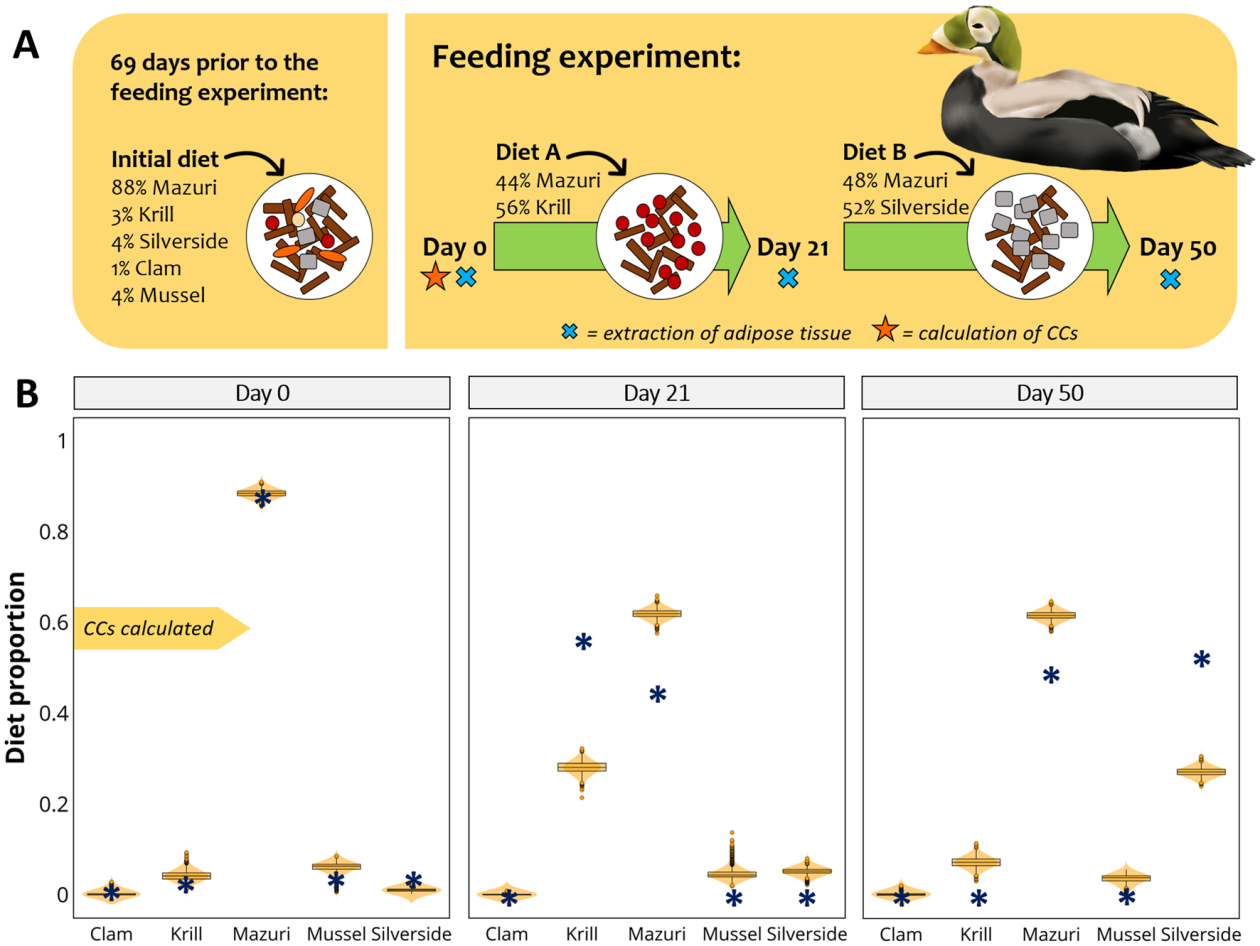
Spectacled eiders case. (**A**) Feeding experiment: spectacled eiders (n = 8) spent 69 days on the initial diet described in the figure; after this, on day 0 eiders were biopsied and switched to diet A. On day 21 they were biopsied again and switched to diet B. After 29 days on diet B, eiders were biopsied on day 50. (**B**) Plots for diet estimations of spectacled eiders fed different combined diets. The true diet is indicated in each plot by the blue asterisks. CCs were calculated using FA data of day 0. Images by Alicia Guerrero.

This case is based on captive feeding trials conducted on 8 adult spectacled eiders, *Somateria fischeri*, which were maintained on an initial diet containing 1% Atlantic surf clam, 3% Antarctic krill, 88% Mazuri sea duck formula, 4% blue mussel and 4% Atlantic silverside, for 69 days prior to the start of the feeding experiment. After this, on day 0, a biopsy sample of the synsacral adipose tissue was obtained from each eider. With the FA data of the adipose tissue the authors calculated CCs. Feeding trials started on Day 0, and spectacled eiders were switched to diet A, consisting of 56% krill and 44% Mazuri sea duck formula for 21 days. On Day 21, eiders were biopsied again and switched to diet B consisting of 48% Mazuri formula and 52% silverside. On Day 50, a final biopsy sample was collected (Fig. 2A). FA turnover was considered near complete by 69 days.

#### The model

This analysis is based on FA data from day 0, 21 and 50. We used the CCs calculated in this study after eiders were maintained on the same initial diet for 69 days. All sources were significantly different from each other (PERMANOVA, *F*_4_ = 9936.9, *P* = 0.001). ‘Day’ was set as fixed factor in the model.

#### Diet predictions

Based on FA data of day 0, MixSIAR estimated a contribution of 0.1% clam, 4% krill, 88% Mazuri, 6% mussel, and 1% silverside (Fig. 2B). After the first shift of diet, MixSIAR estimations changed to 28% krill and 62% Mazuri for FA data obtained on day 21. The final biopsy sample on day 50 produced estimations of 62% Mazuri, and 27% silverside.

### Case 2: Steller’s eiders, from Wang et al***.***^42^

#### The experiment

**Figure 3:**
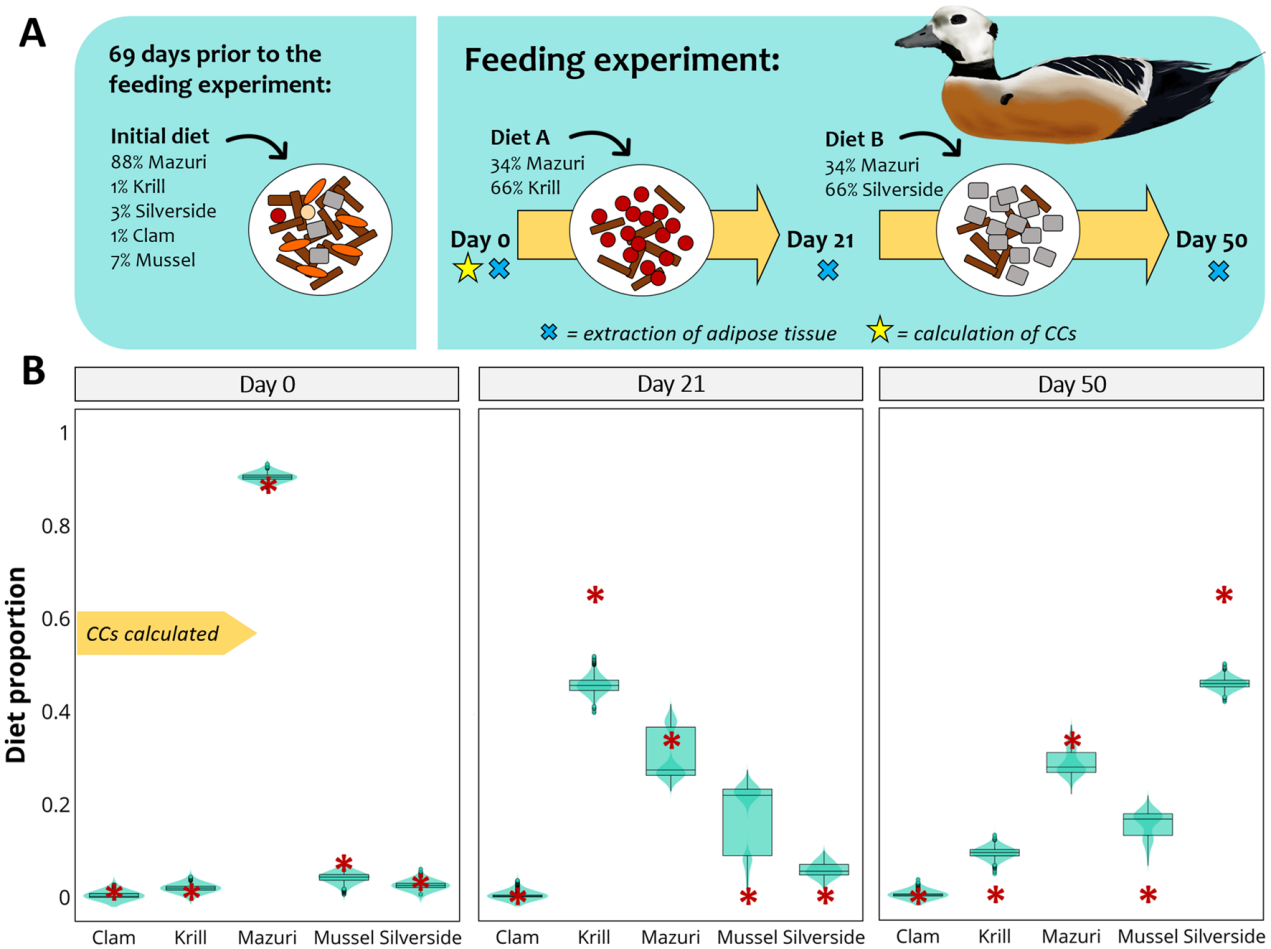
Steller’s eiders case. (**A**) Feeding experiment: eiders (n = 8) were maintained on the initial diet described in the figure for 69 days after which biopsy samples were collected (day 0) and eiders were switched to diet A. After 21 days, eiders were biopsied again and switched to diet B. On day 50, after 29 days on diet B, eiders were biopsied one last time. (**B**) Plots of diet estimation for Steller’s eiders fed different combined diets. Diet estimates are based on biopsy samples collected on days 0, 21 and 50. The true diet is indicated in each plot by red asterisks. CCs were calculated using FA data of day 0. Images by Alicia Guerrero.

This case corresponds to a feeding trial conducted simultaneously to the previous case by Wang et al*.*^42^, although the diet of Steller’s eiders, *Polysticta stelleri*, differed slightly. For 69 days prior to the start of the feeding trial, 8 adult Steller’s eiders were maintained on an initial diet containing 1% clam, 1% Antarctic krill, 88% Mazuri sea duck formula, 7% mussel, and 3% silverside. CCs were calculated after a biopsy was extracted to each eider on day 0. At the start of the feeding experiment, on day 0, Steller’s eiders were switched to diet A, containing 66% krill and 34% Mazuri formula. Then, on day 21, they were switched to diet B, consisting of 34% Mazuri formula and 66% silverside. Biopsy samples were collected on days 0, 21 and 50 (Fig. 3A). FA turnover was considered near complete by 69 days.

#### The model

We used the CCs calculated in the same study after Steller’s eiders were maintained on the same diet for 69 days. All sources were significantly different from each other (PERMANOVA, *F*_4_ = 10,079, *P* = 0.001). ‘Day’ was set as fixed factor in the model.

#### Diet predictions

Based on FA data of Day 0, MixSIAR estimated a contribution of 0.2% clam, 2% krill, 90% Mazuri, 4% mussel, and 3% silverside (Fig. 3B). After the first diet switch, MixSIAR estimations changed to 46% krill and 27% Mazuri. The final biopsy sample on day 50 produced estimations of 9% krill, 28% Mazuri, and 46% silverside.

### Case 3: Atlantic salmon, from Budge et al***.***^43^

#### The experiment

**Figure 4:**
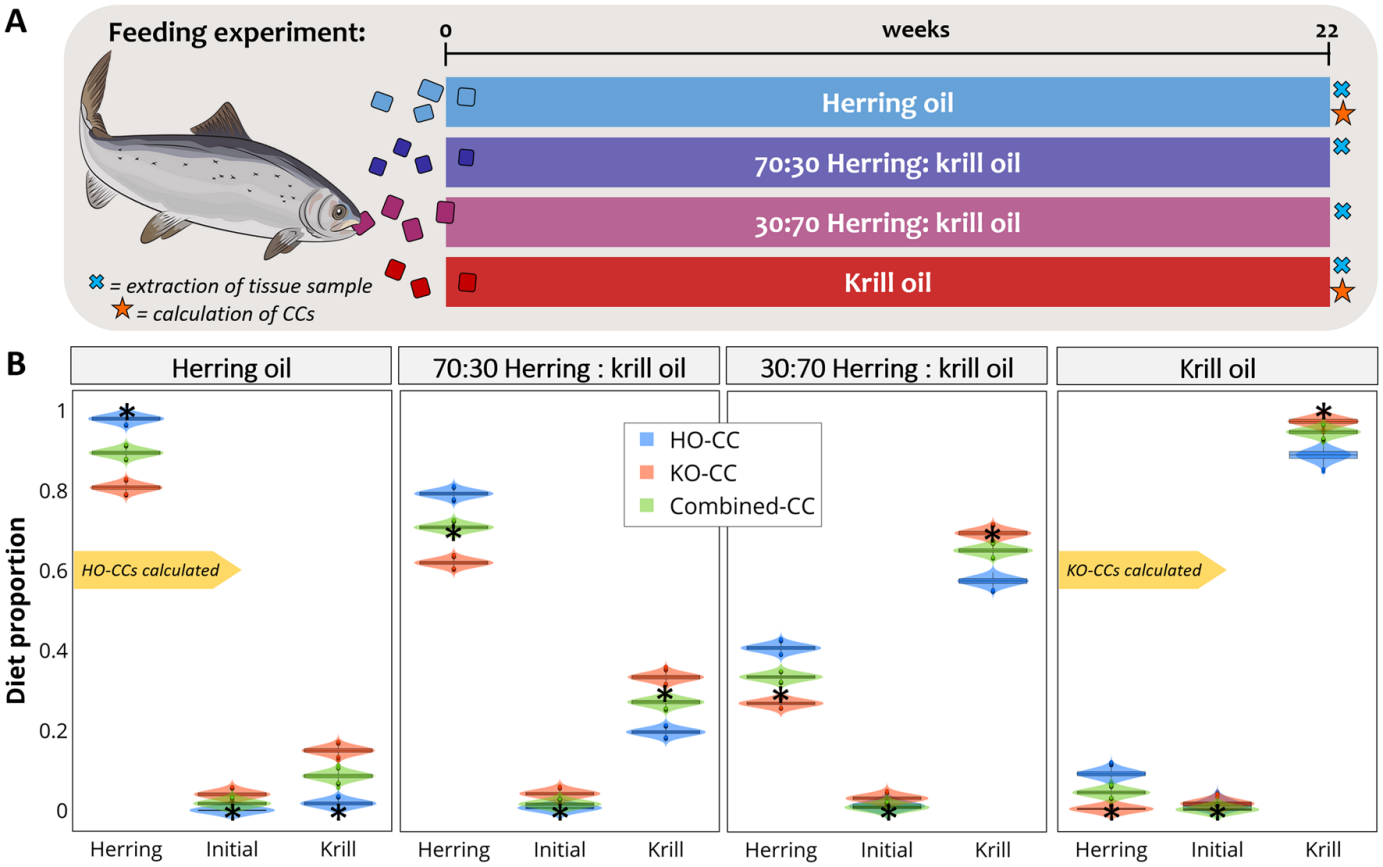
Atlantic salmon case. (**A**) Feeding experiment: Atlantic salmon fed formulated feeds based on either solely herring (n = 36) or krill oil (n = 28), or in proportions of 70:30 or 30:70 herring to krill oil (n = 34 each), for 22 weeks. (**B**) Plots of diet proportions estimated using MixSIAR for models using CCs derived from salmon fed a herring-oil diet (HO-CC), a krill-oil diet (KO-CC) or CCs averaged from these two treatments (Combined-CC). The true diet is indicated in each plot by black asterisks. Salmon image designed by Creazilla (https://creazilla.com).

For 22 weeks, tank-reared juvenile Atlantic salmon, *Salmo salar* (n = 132)*,* were fed one of four different formulated feeds based on two marine oils: 100% krill oil, 100% herring oil, a mixture of 70:30 herring to krill oil, or a mixture of 30:70 herring to krill oil. Muscle samples were analysed for FAs after the 22-week experiment, which allowed the calculation of CCs. In this experiment, two sets of CCs were calculated: one derived from salmon fed the diet based on 100% herring oil and another from salmon fed the diet based on 100% krill oil (Fig. 4A). Unlike the previous two cases, where CCs were derived from consumers eating a mixed diet, here CCs were obtained from consumers feeding on a single type of source: either herring or krill oil. This allowed us to evaluate whether the source used to calculate the CCs affected dietary predictions. Additionally, we calculated a combined CC (an average value between CCs derived from krill and herring diets) and run a separate model. FA turnover was considered complete after 22 weeks on the same diet.

#### The model

We ran three independent models to estimate the diet of salmon fed each of the four diets: one using the set of CCs derived from herring oil, another using the CCs derived from krill oil, and one using the combined CCs. For each model, we used krill oil, herring oil and initial diet (commercial feed given to salmon prior to the experiment) as sources, and set ‘diet group’ as fixed factor. Significantly different FA compositions were found for the three sets of sources: those multiplied by herring oil CCs (PERMANOVA, *F*_2_ = 4008.7, *P* = 0.003), those multiplied by krill oil CCs (PERMANOVA, *F*_2_ = 3354.7, *P* = 0.002), and those multiplied by the combined CCs (PERMANOVA, *F*_2_ = 3677.2, *P* = 0.003).

#### Diet predictions

When we used CCs derived from salmon fed on a diet supplemented with herring oil only, MixSIAR correctly estimated the contribution of herring oil in the consumers (salmon) diet (98%). However, the contribution of herring was slightly overestimated where the consumers had been fed a mixture of herring and krill oil (Fig. 4B), and where the salmon’s diet was based on krill oil the contribution of krill oil was slightly underestimated (89%).

We found the opposite trend when we used CCs derived from salmon that had been fed a diet where krill oil had been the lipid source. Here, MixSIAR correctly estimated the contribution of krill oil in the salmon’s diet when they had been fed a diet based on krill oil (98%) or a mixture of 70:30 herring to krill oil (33% krill) or 30:70 herring to krill oil (70% krill); however, when herring oil had been the only dietary source this dietary contribution was underestimated (81%) (Fig. 4B).

Our dietary estimates were less biased when we used CC values that had been derived from the average between the herring- and krill-oil treatments (Combined-CCs). For example, we estimated herring contribution to be 90% of the diet when the actual diet was supplemented only with herring oil, and an estimate of 95% krill contribution when the actual diet was supplemented with krill oil only, and when the actual diet was a combination of herring (70%) and krill (30%), the estimations were 71% and 27%, and where the actual contribution of herring was 30% and 70% of krill, the estimated diets were 34% herring and 65% krill (Fig. 4B).

### Case 4: tufted puffin nestlings, from Williams et al. ^44^

#### The experiment

**Figure 5:**
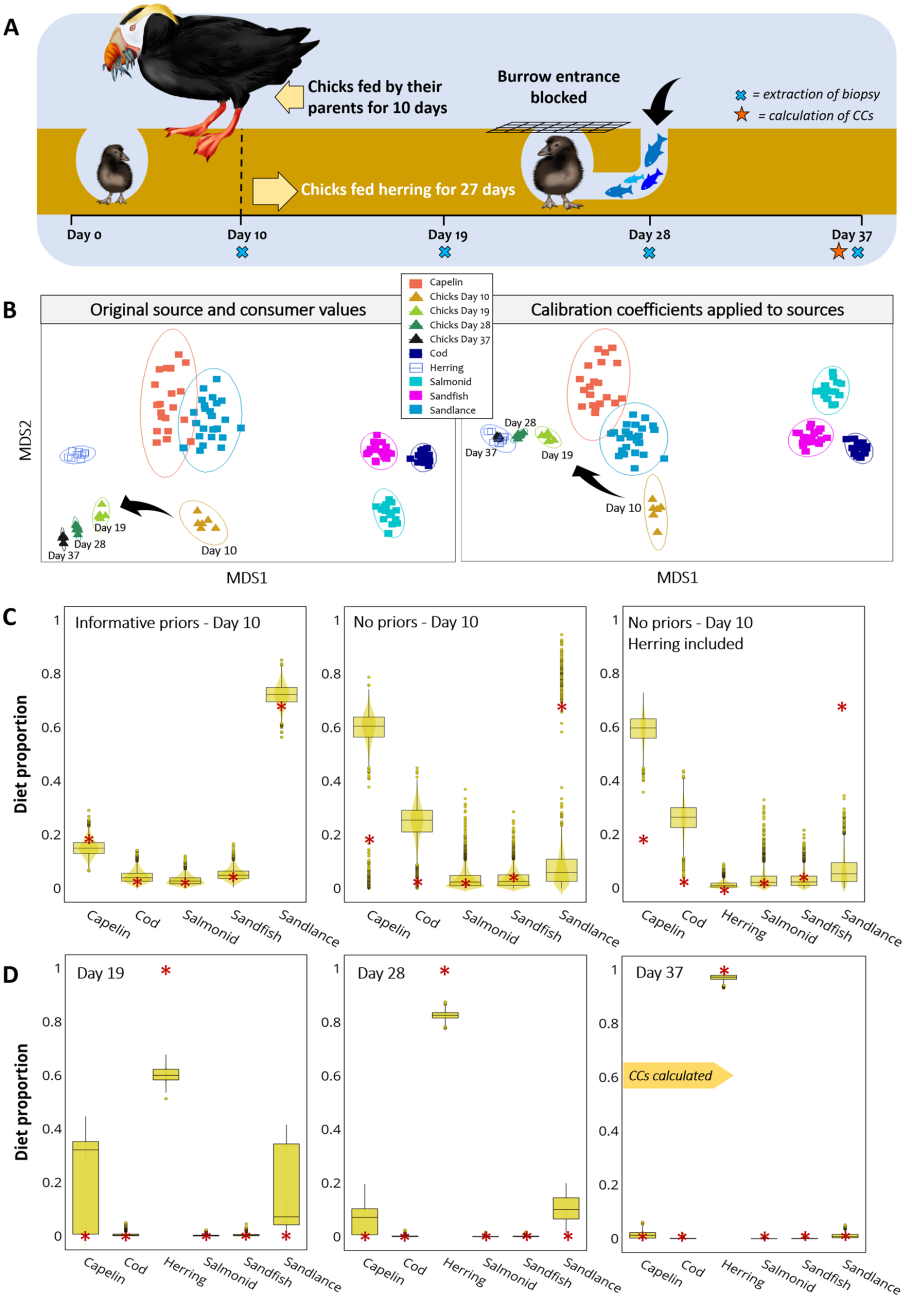
Tufted puffins case. (**A**) Nestlings (n = 6) were fed by their parents for approximately 10 days since hatching. After this, they were fed herring for another 27 days as the entrance to their burrows was blocked and parents could not feed their chicks. (**B**) Non-metric multidimensional scaling plots for FAs obtained from chicks at different stages of the experiment and their sources. When FAs of sources were multiplied by their respective CCs, source (herring) and consumer (chicks, day 37) overlap in the plot. (**C**) Plots of the three models run to estimate the diet of nestlings on day 10. From left to right: Model using informative priors based on meals brought by the parents after the burrows were blocked; the same model without informative priors; and a third model without informative priors but including herring as source even though it was not part of the nestlings’ diet. The red asterisks in each plot represent the potential diet fed by the parents (and used as priors in the first model). **(D**) Plots of diet estimations for tufted puffins fed herring, based on their FA profiles of days 19, 28 and 37. The true diet is indicated in each plot by red asterisks. CCs were calculated from tufted puffins fed herring, using FAs from day 37. Images by Alicia Guerrero.

Tufted puffin, *Fratercula cirrhata*, nestlings (n = 6) underwent an experimental feeding trial in their own burrows. Chicks were fed by their parents for approximately 10 days since hatching. When they were estimated to be 10-days old, the access to the burrows was blocked, so adults could not continue feeding their chicks. Through another access hole excavated by the researchers, chicks began being fed Pacific herring once a day, for 27 days. To infer the diet of free-living puffin nestlings during the first 10 days after hatching, wire screens were placed at burrow entrances to collect whole fish dropped by the parents. The species identified, in descending order (by mass), were Pacific sandlance, capelin, Pacific sandfish, salmonid and Pacific cod. An adipose tissue sample was collected on days 10 (start of the feeding trial), 19, 28 and 37. On day 37, assuming complete FA turnover after 27 days on a single prey diet (herring), the researchers calculated CCs (Fig. 5A). The data used to run this model included day 10, which represents the unknown diet provided by the parents, days 19, 28, and 37 which represent the herring diet at different extents. FA turnover was considered “close to, but not entirely complete” after 27 days^44^.

#### The model

We used the CCs derived from these chicks feeding on Pacific herring for 27 days. For biopsy samples collected on day 10, we conducted three separate analyses: the first model excluded herring as potential prey, and incorporated informative priors based on the amount of different fish (% by mass) dropped by the parents at the burrow entrances; the second model was exactly the same but without prior information; and the third model was run without priors, and included herring as potential prey in order to determine whether this prey could be identified as absent.

For days 19, 28 and 37, we ran another analysis and included herring as source and no informative priors. ‘Day’ was set as a fixed factor in this model. All sources had significantly different FA compositions (PERMANOVA, *F*_5_ = 430.9, *P* = 0.001).

#### Diet predictions

In this example we included a non-metric dimensional scaling plot (Fig. 5B) to evaluate the effect of applying CCs to sources. Day 0 biopsies show greater variation than those of successive days, in both plots. When using original sources and consumer FA values, chicks are segregated from all the sources, although the similarity of the FAs increases toward the FA of herring as days pass, but they do not match. When CCs were applied to sources, the FA values of herring and chicks from day 37 overlap, indicating that they have the same FA compositions.

For day 0 (Fig. 5C), the estimated diet contributions were similar to meals brought by the parents when the model included informative priors, where the main dietary sources were sandlance (72%) and capelin (15%). Whereas estimates from the model without informative priors misrepresented the diet, as capelin was wrongly identified as the main dietary source (61%), cod the second most important prey (26%), and sandlance was incorrectly estimated to be only 6% of the diet. The third model including herring again identified capelin and cod as the main contributors (56% and 23%, respectively) whereas herring was identified as the least important prey, with 0.9% of contribution.

For day 19 (Fig. 5D), herring was identified as the main source, with 60% of contribution, followed by capelin and sandlance although with greater variation. From day 19 to 37, the contribution of herring increases from 60 to 97%, respectively.

### Case 5. Harp seals, from Kirsch et al***.***^45^

#### The experiment

**Figure 6:**
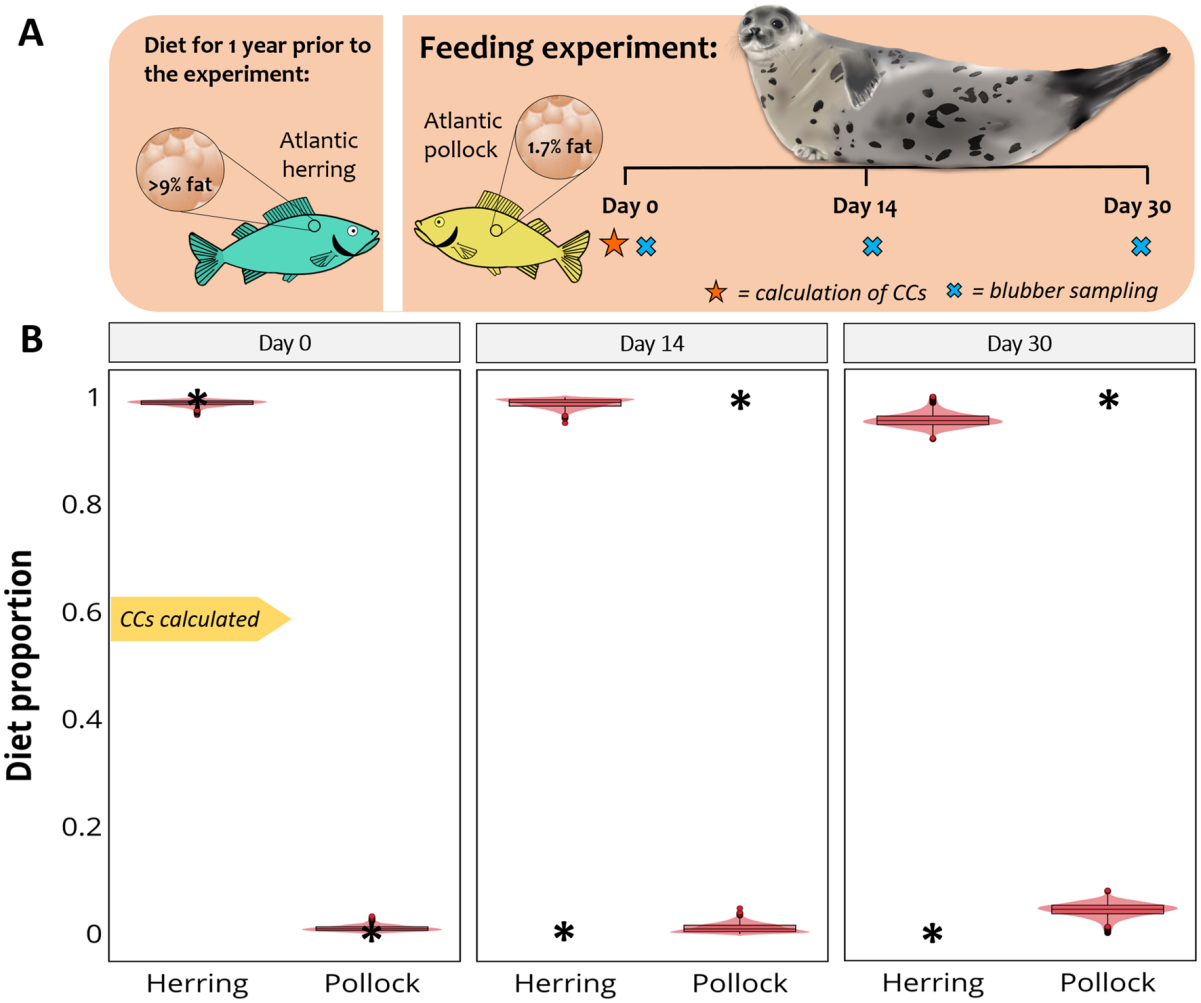
Diet estimates for juvenile harp seals fed a low-fat prey. (**A**) Feeding experiment: For a year prior to the feeding experiment, harp seals (n = 5) had been eating only Atlantic herring, a prey with a high-fat content. During the feeding experiment, seals were fed Atlantic pollock, a low-fat prey, for 30 days. (**B**) Plots of estimates derived from MixSIAR models based on FA data of whole blubber cores from days 0, 14 and 30. The black asterisks in the plots indicate the true diet. CCs correspond to harp seals fed herring, calculated using FAs from day 0. Images: fish by Lukas Guerrero Zambra, harp seal by Alicia Guerrero.

This study evaluated the effect of a low-fat diet on blubber FAs of harp seals, *Pagophilus groenlandicus*. Only for this experiment, the fat content of the different sources was available. Juvenile harp seals (n = 5) had been maintained on a diet of Atlantic herring (≥ 9% fat) for approximately 1 year prior to the feeding trial. On day 0, a full-depth blubber sample was collected from the posterior flank of each animal. For 30 days, seals were kept on a diet consisting solely of Atlantic pollock (1.7% fat). Blubber biopsies were taken again on days 14 and 30 (Fig. 6A). FA turnover was not considered complete after 30 days on the same diet, and authors suggest that a longer period on the diet, or higher intakes of fat, would be needed to accomplish it.

#### The model

Since seals had been feeding on the same source for a year, we calculated CCs using FA data from day 0. Thus, consumer values were divided by Atlantic herring values producing CCs that were applied to both herring and pollock FA data. Sources were significantly different (PERMANOVA, *F*_1_ = 3680.4, *P* = 0.001) and ‘day’ was set as fixed factor in the model.

#### Diet predictions

For all three data sets (day 0, 14 and 30) the main contributor to the diet was Atlantic herring. The predicted proportion of Atlantic herring decreased only slightly from 99% on day 0 to 95% on day 30. Consequently, the contribution of pollock increased from 1% on day 0, to 5% on day 30 (Fig. 6B).

### Case 6: Harbour seals, from Nordstrom et al.^46^

#### The experiment

Estimations are based on a feeding experiment by Nordstrom et al*.*^46^. Prior to the feeding study, juvenile harbour seals, *Phoca vitulina*, were fed a homogenate of 2:1 Pacific herring to salmon oil for approximately three weeks, and then fed only Pacific herring for four to six days. The feeding experiment consisted of three diets: one group of seals was fed only herring for 42 days (n = 3); the second group was fed only surf smelt for the same period (n = 6); and a third group (n = 7) was fed smelt for 21 days and then only herring for 21 days (Fig, 7A). Whole blubber core samples were collected on days 0, 21 and 42 for each group. Complete FA turnover was estimated to occur after at least 55 days on the same diet, although it could extend well beyond if turnover rate slowed with time.

#### The model

Since prey FA data was not provided in the same study, we used Pacific herring, surf smelt, and salmon FA values from Huynh and Kitts^47^, which had significantly different FA composition (PERMANOVA, *F*_2_ = 87.7, *P* = 0.001). CCs were derived from other harbour seals on a Pacific herring diet for over a year, in the same study^46^. We estimated the diet of the three groups of harbour seals, based on samples collected on day 42, setting ‘diet group’ as fixed factor in our model.

#### Diet predictions

**Figure 7:**
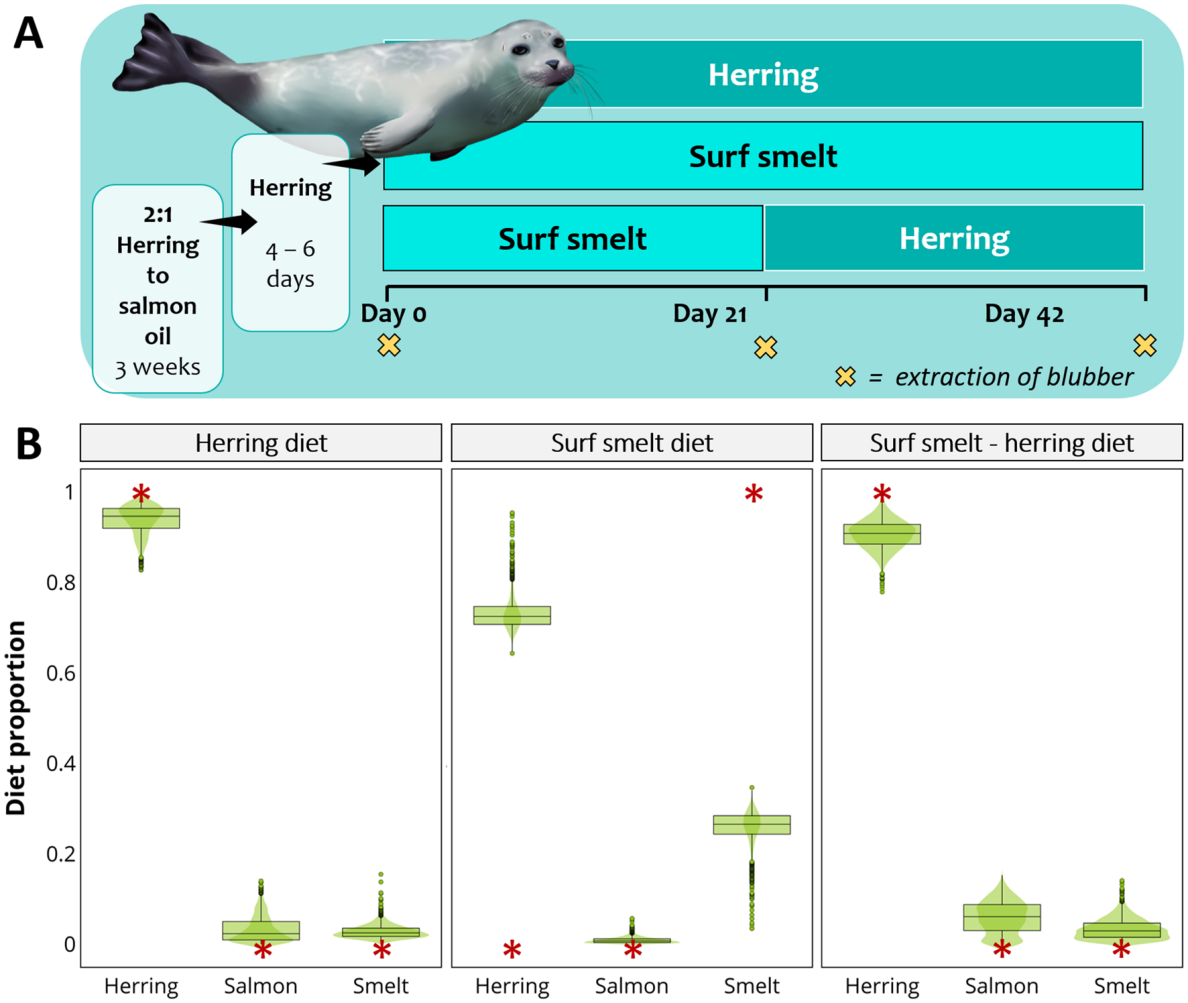
Diet estimates for harbour seals. (**A**) Feeding experiment: For 42 days, seals were fed the following diets: solely herring (n = 3), solely surf smelt (n = 6), or surf smelt for the first 21 days and then herring for the remaining 21 days (n = 7). Prior to the feeding experiments they had been fed a mixture of herring and salmon. (**B**) Plots for MixSIAR diet estimations, based on blubber FAs obtained on day 42. The red asterisks indicate the true diet. CCs were obtained from harbour seals (other individuals) fed herring for over a year. Image by Alicia Guerrero.

Overall, diet differences were evident among groups, and the direction of the change was consistent with the shifts in diet. Estimates for harbour seals fed exclusively Pacific herring for 42 days, correctly showed that diet was predominantly based on herring (94.7%). For seals fed surf smelt for 42 days; however, estimates showed that surf smelt only accounted for 26.6% of the diet whereas herring remained to be the main component. For seals fed surf smelt for 21 days and then herring for another 21 days, MixSIAR again identified herring as the main component, with 90.9%, whereas surf smelt was only 3% (Fig. 7B).

## Discussion

Simulations based on real FA data derived from feeding studies allowed us to evaluate the performance of MixSIAR under a variety of scenarios. Although not all cases provided accurate diet estimates, MixSIAR correctly identified the main dietary components and changes in diet. Although the FA turnover of consumers was not always known, and therefore a quantitative evaluation of MixSIAR was not possible, our results support the use of MixSIAR with proportional FA data for ecological studies.

There is considerable uncertainty regarding the trophic modification of FAs and this is probably the weakest aspect in the application of Bayesian mixing models to FA data. CCs are a basic calculation that tries to describe the complex biochemistry behind trophic modification; however, they are to date the only means we have for accounting for consumer metabolism^42^. In this regard, MixSIAR accurately estimates known diets when CCs were calculated from the same data used in the model (same consumer eating the same sources used to calculate CCs). CCs are calculated by dividing the FA data of the consumer by that of the feed (Fig. 1), and when we take sources to the predator space (prior to modelling) we conduct the opposite mathematical operation (multiply sources by CCs), producing source FA values that are exactly equal to those of the consumer. This is evident in Fig. 5B, where herring FA values match those of tufted puffins from day 37. Consequently, MixSIAR estimates are expected to match the actual diet. This is the case of salmon fed either solely krill or herring (Fig. 4B), tufted puffins on day 37 (Fig. 5D), and harp seals on day 0 (Fig. 6B). The very minor differences between the estimated and the actual diets may be due to noise introduced by creating random values from means and standard deviations of the original FA values.

In the case of spectacled and Steller’s eiders, the CCs were calculated using a mixed diet and not a single diet source. Nevertheless, MixSIAR produced diet estimations that closely resemble the actual diet (Figs. 2 and 3). Although, a better evaluation of the performance of MixSIAR would be to use CCs not calculated from the same data. As expected, the best dietary predictions were obtained with the FA data used to calculate the CCs (day 0); however, estimates over successive days followed the direction of the diet switch. Our results for the spectacled eiders were similar to those of the original study, where Wang et al.^42^ used the QFASA method. For spectacled eiders, samples collected on day 21 showed an increase in the contribution of krill and decrease in Mazuri formula, resembling the diet given to the eiders for the 21 days prior to the biopsy collection. The proportions of each source were not expected to equal their actual diet, since 21 days is insufficient for complete FA turnover; therefore, their adipose tissues would still reflect part of their diet prior to the feeding study. A similar pattern was observed with the samples collected on day 50, which was only 29 days after the second diet shift, and where the contribution of silverside increased but not as much as in the actual diet. Our results for Steller’s eiders were closer to the true diet than those obtained with QFASA in the original study. The contribution of mussel, however, was overestimated on days 21 and 50, and showed a bimodal distribution by day 21. In the original study, QFASA also overestimated the proportions of mussel when the model was run using extended dietary FAs, but was more accurate with reduced FA subsets. These examples suggest that MixSIAR produces correct diet estimations, even when the CCs have been calculated based on a different diet (initial diet). However, a longer period is needed for eiders to replace their FAs completely, which coincides with the conclusions drawn by the authors of the original study^42^. Unfortunately, the uncertainty regarding the FA turnover prevents us evaluating the performance of MixSIAR in a quantitative manner; and this is a serious limitation.

The best-case scenario, is to incorporate into the models not only the FA data for the consumers, but those of potential prey items, as well as CCs derived under controlled conditions (i.e., feeding trials), this however, will not always be possible, particularly when studying wild populations in remote regions. The use of CCs, or trophic modification values, are an important consideration for quantitative diet estimation, regardless of the statistical method selected. It is proposed that when controlled feeding experiments are not feasible, an alternative is to use CCs (or trophic fractionation factors, in the case of stable isotopes) derived from related species, or the same species fed on a different diet. Although, caution needs to be highlighted, as FA data for CCs that have been obtained from captive animals may differ from their wild counterparts, and CCs can vary depending on the diet the test subjects were fed^48,49^. Although, dietary predictions were reasonable even when using trophic modification values (CCs) derived from different prey, the model performance improves when we use CC values averaged across single-diet studies. In our study with the Atlantic salmon as consumers, where we used CCs derived from a herring-oil diet we tended to overestimate the contribution of herring oil in the salmons’ diet. However, where we used the CCs derived from a krill-oil diet, our estimates of the salmon’s diet were closer to their actual diet, although if salmon had been fed only herring oil, it was underestimated in their diet. Our findings are similar to those of Rosen and Tollit^50^ using QFASA, where they found that when using CCs derived from seals fed herring, the model predicted the amount of herring consumed more accurately than when they used CCs derived from seals fed eulachon. Similarly, dietary predictions overestimated the contribution of eulachon, when the CCs were derived from seals fed eulachon.

Interestingly, although we find some differences where we use CCs derived from different prey, overall, the prey composition estimates were similar to the true diet. This was also found in the study that used QFASA to estimate dietary composition^43^. Similarly, in our harbour and harp seal cases, CCs were calculated using herring and, in both cases herring was overestimated compared to the true diet. Since mixing models are sensitive to variation in trophic fractionation values^51^, the specificity of CCs strongly influences model performance. Indeed, the trophic modification of the biological tracer (i.e., CC’s for FA data) between the prey’s tissue and incorporation within the consumers’ tissue, remains the weakest aspect in the ability of mixing models to reconstruct diet^51,52^. This is not unique to modelling with FA data, as it is true also for modelling with all biochemical tracers (i.e., stable isotopes), and all diet-estimation methods. Bond et al*.*^51^ showed that varying trophic discrimination factors changed substantially the dietary estimates produced by SIAR with stable isotope data. We show, with our Atlantic salmon case, that the same is true when modelling FAs, as our diet estimations, although not absolutely accurate, were less biased where we averaged CCs across values derived from two treatments where consumers had been fed single-prey diets than if we used a single, prey-specific CC. Another alternative to using single-prey CC’s could be to use CCs calculated from consumers fed on mixed diets, rather than on one-prey diets only.

Brett et al*.*^33^ state that it is critical to measure and directly account for trophic fractionation for all the major food sources. One way to implement this method is to use the FAs of consumers fed various single-prey diets instead of the FAs of the actual sources. The development of resource libraries; however, is time consuming, economically costly, limited to animals relatively easy to manipulate in captivity^48^, and furthermore, the FA profiles of captive animals might not be comparable to their free-living counterparts. Since this is an important limitation of diet-estimation methods, other modelling approaches have been developed to estimate trophic modification values. The R package SIDER, for example, estimates trophic discrimination factors of stable isotopes based on tissue type, feeding ecology and phylogenetic information of the consumer. Regardless of the method used to account for trophic modification, the diet estimation process can benefit from the use of additional information, such as field observations, or other complementary methods^51^.

The case of tufted puffins allowed us to evaluate how the addition of prior information can improve diet estimates. The model with prior information was very similar to the proportions of meals brought by the parents (with sandlance as the main prey), suggesting that the priors have great influence on the outcomes. The model without priors selected capelin as the main prey item, whereas sandlance was not a very important prey. These two species had similar FA compositions (Fig. 5B), and although they did differ statistically, their overall similarity could confound the model resulting in numerous outliers in their posterior distributions (Fig. 5C). If this is the case, informative priors could be particularly helpful to distinguish sources with similar tracer values. Litmanen et al.^30^ suggest that if prior information is available, MixSIAR is recommended to estimate diet over other methods. We, therefore, recommend the use of MixSIAR based on FA data in complement with other methods such as stomach or faeces content analysis, direct observation, and/or stable isotopes.

The tufted puffin case also allowed us to test whether a source could be identified as absent. In Bayesian models none of the diet proportions can be zero, therefore a very low estimated proportion can also indicate an absent of a source. In our example, there was a small proportion of herring estimated (i.e., 0.009 or 0.9%) which was the smallest proportion of all the sources. Litmanen et al*.*^30^ found that both Bayesian and QFASA methods had more problems identifying absent sources for herbivorous zooplankton when the number of absent sources was higher; however, there were exceptions. Although here MixSIAR did produce a small estimate for herring, more systematic testing is needed in order to understand when an absent source can be correctly identified. Compared to the fish and bird studies our models with FA data were less accurate at estimating the diet of the mammal species in our study, the harbour and the harp seal. There are several possible factors, associated with both metabolism and model settings, that may explain these differences. The mammals were marine mammals, and have stratified blubber, where the FAs vary in the position along the blubber core^16,53,54^. The transverse variation in FAs across the blubber has been attributed to the different roles that the outer and inner blubber layers play^55–57^. The FAs of the inner blubber layer have an active turnover rate as they are more directly influenced by the consumer’s diet^56,58,59^ while the FAs in the outer blubber play an active role in thermoregulation^55^, they are more stable and less affected by short-term dietary changes^55,60,61^. For example, Struntz et al*.*^61^ observed that the outer layer of bottlenose dolphins, *Tursiops truncatus*, remained unchanged after a period of undernourishment. Similarly, fin whales, *Balaenoptera physalus*, did not obtain their energy reserves from the outer layer during pregnancy^62^. Guerrero and Rogers^55^ found that the FAs of the outer blubber of pinnipeds in cold higher latitudes have increased desaturation in line with the outer blubbers’ increased thermoregulatory role. The FA data we used for our two seal cases were derived from full-depth blubber cores, so that this data included FAs from the outer blubber, where FAs are more stable. This means that although the FAs from the feeding trial diet were likely to be adopted into the inner blubber layer they were less likely to have been assimilated into the outer blubber layer, where FAs are very stable. Our study pooled the FA results from across the blubber layers; the effect of incorporating FA values from both the inner and outer layers of the blubber may have dampened the ability to detect the FAs due to the dietary changes in the feeding trial^63,64^. The group of harbour seals fed surf smelt showed the highest estimated contribution of this prey of the three groups, whereas the group fed both surf smelt and then herring was estimated to consume lower proportions of surf smelt than the group fed solely smelt, but higher proportions than the group fed solely herring. Thus, the estimated diet proportions moved in the same direction as the diet change, and in concordance with what we know about how diet influences blubber FAs. It is worth noting that for this case, FA values of the potential sources were obtained from the literature, and probably differ at some degree from the actual prey used to feed the seals, which likely also affected the outcomes.

For consumers fed on low-lipid diets, such as our harp seal case, there may be a bias in diet estimates. Lipids are stored in the blubber only when food intake is greater than their energy expenditure^65^ so that where an animals’ diet does not meet maintenance energy needs, they may not store dietary lipids^44^. Thus, in our case, harp seals could be using all the energy derived from the pollock for their daily metabolic processes, in which case the FAs in the blubber would not reflect recent dietary FAs, since there would have been little or no surplus energy (FAs) to be stored. In fact, Kirsch et al*.*^45^ simulated the FA composition of harp seals under three scenarios: where 100%, 50% or 25% of the FAs from diet were deposited in the blubber. They found that the final FA composition of harp seals on day 30 was more like the scenario where 25% of the FA had been deposited, suggesting that their blubber did not closely reflect their current diet. Therefore, for both our seal cases, physiological factors were likely to have influenced FA turnover. Thus, although these cases still support the use of MixSIAR with FA data to estimate diet, we recommend caution is exercised in interpreting the results with particular attention to the potential effect of nutritional state on lipid metabolism^44^. Diet estimation based on FA data represents information stored in the adipose tissues of the consumer and, therefore, it is dependent on the fat content of each source. Models could give fat-rich prey a greater contribution due to their high fat content. This can be accounted for in the diet estimation model by incorporating “concentration dependence”^28^. We suggest taking this into account when performing quantitative dietary studies.

A limitation of our study is the use of simulated FA data due to the unavailability of the consumers’ raw FA data. The simulated data may not act like actual FA profiles, which are correlated. Our simulated data, however, although not correlated for a single individual, are compositional for the entire sample (i.e. the summed FAs do co-vary). Therefore, since our models were conducted for the whole sample each time, and not for a single individual, this should not be a problem. As detailed in the Supplementary Material 1, the comparison between simulated and actual FA data produced the same diet estimates for wild squids.

The cases tested show that this Bayesian mixing approach is useful for FA data, and we suggest that if used to complement other dietary methods, for example FA and stable isotope data in parallel, it has potential to explain trophic interactions^25^. The use of FA data could improve taxonomic resolution when used alongside stable isotope data. Unlike stable isotope analyses, where usually only two variables are used (e.g., δ^13^C and δ^15^N), there are a great number of FAs in animals’ tissue (i.e., > 20^25^), which facilitates species discrimination. Where closely related source species, such as fish, can have similar stable isotope values, commonly they have different FA compositions. This emphasizes the potential of using FA data with this Bayesian framework. We encourage the use of FA as quantitative tracer alongside other methods to help improve diet estimations.

## Supplementary information

Supplementary Information 1.

Supplementary Information 2.

Supplementary Information 3.

## Data Availability

All data supporting the conclusions of this article are within the paper and the supplementary materials.
