# Supplementary material for: Evaluating the performance of the Bayesian mixing tool MixSIAR with fatty acid data for quantitative estimation of diet
Source: Sci Rep. 2020 Nov 27;10:20780. doi: 10.1038/s41598-020-77396-1 (PMC7695706; doi:10.1038/s41598-020-77396-1)
Supplement: Supplementary file 1 — Supplementary Information 1. [file 41598_2020_77396_MOESM1_ESM.docx]

**Evaluating the performance of the Bayesian mixing tool MixSIAR with fatty acid data for quantitative estimation of diet**

Alicia I. Guerrero^a^ and Tracey L. Rogers^b^

**SUPPLEMENTARY MATERIAL 1**

^a^ Centro de Investigación y Gestión de Recursos Naturales (CIGREN), Instituto de Biología, Facultad de Ciencias, Universidad de Valparaíso. Gran Bretaña

1111, Playa Ancha, Valparaíso, Chile.

^b^ Evolution and Ecology Research Centre, School of Biological, Earth and Environmental Sciences, University of New South Wales, Sydney, Australia 2052

*Corresponding author:

Alicia I. Guerrero

alicia.guerrero@uv.cl

**Use of simulated fatty acid data versus original fatty acid profiles**

We assessed the effect of using simulated data derived from the means and standard deviations, instead of using actual fatty acid profiles. To do this, we estimated the diet of the squid *Lolliguncula brevis*, using data from Stowasser et al. 2006 (made available in Happel et al. 2017). In the original study, squids were fed on prey species with different fatty acid profiles over 30 consecutive days. From this experiment, we used the group “wild squid” (with unknown diets) as consumers, and the squid groups fed either crustaceans or fish, as sources. CCs were set to zero, as squid fed single diets for 30 days already incorporated CCs.

The raw fatty acid data of each consumer (Table S1) was available in Happel et al (2017); we used these original data to run our first model. We then calculated the means and standard deviations of each fatty acid, and generated random values (Table S2) with the function ‘rnorm’ in R. This was done for each one of the fatty acids of wild squids, thus, we run our second model using these simulated consumer data.

Using the posterior distributions of each model, we run *t*-tests in order to determine whether diet estimations obtained from simulated and original consumer fatty acid profiles differed.

In both models, MixSIAR estimated that wild squid consumed mainly fish. The original data produced a diet estimation of 94.0% fish and 6.0% crustaceans whereas the simulated data produced estimations of 94.1% fish and 5.9% crustaceans (Table S3). There were no significant differences in the contributions estimated either for crustaceans (*t* = -0.34, *df* = 5998, *P* = 0.731) or fish (*t* = 0.34, *df* = 5998, *P* = 0.731).

In this study all the examples have been conducted using simulated data, due to the unavailability of raw fatty acid data in the studies selected. This analysis shows that the simulation of fatty acid profiles should not provide different results compared to actual fatty acid profiles. This is because diet contributions are calculated for the whole sample (e.g. 5 wild squids) and not for each consumer individually.

**Table S1.** Original fatty acid profiles of wild squid, from Stowasser et al. (2006)

| **Diet** | **Wild** | **Wild** | **Wild** | **Wild** | **Wild** |
| --- | --- | --- | --- | --- | --- |
| **C14.0** | 2.01 | 0.92 | 2.33 | 1.17 | 1.39 |
| **C16.0** | 21.78 | 20.12 | 19.77 | 18.9 | 17.58 |
| **C16.1n7** | 2.52 | 1.19 | 2.64 | 1.11 | 2.56 |
| **C18.0** | 11.2 | 11.25 | 10.3 | 12.83 | 11.9 |
| **C18.1n9** | 2.96 | 3.02 | 5.54 | 2.58 | 4.91 |
| **C18.1n7** | 2.18 | 2.2 | 2.32 | 1.99 | 3.12 |
| **C18.2n6** | 0.39 | 0.55 | 0.62 | 0.32 | 1.2 |
| **C18.3n3** | 0.46 | 0.28 | 0.4 | 0.21 | 0.96 |
| **C20.1n11** | 1.22 | 2.17 | 1.9 | 1.32 | 1.36 |
| **C20.1n9** | 0.3 | 0.26 | 0.41 | 0.23 | 0.28 |
| **C20.4n6** | 3.4 | 5.34 | 4.19 | 4.53 | 6.07 |
| **C20.5n3** | 17.7 | 16.77 | 17.35 | 19.41 | 17.29 |
| **C22.1n11** | 0.25 | 1.09 | 0.73 | 0.07 | 0.17 |
| **C22.5n3** | 1.9 | 1.23 | 1.2 | 1.05 | 1.45 |
| **C22.6n3** | 28.87 | 29.52 | 26.11 | 31.81 | 27.87 |
| **C24.1n9** | 0.64 | 0.86 | 1.73 | 0.84 | 0.05 |

**Table S2.** Simulated data, generated using the means and standard deviations of each fatty acid of wild squids, from Stowasser et al (2006).

| **Diet** | **Wild** | **Wild** | **Wild** | **Wild** | **Wild** |
| --- | --- | --- | --- | --- | --- |
| **C14.0** | 1.16 | 2.4 | 1.74 | 1.65 | 0.87 |
| **C16.0** | 19.32 | 18.78 | 20.41 | 21.84 | 17.8 |
| **C16.1n7** | 2.65 | 1.73 | 2.98 | 1.1 | 1.56 |
| **C18.0** | 11.48 | 11.68 | 12.5 | 11.85 | 9.97 |
| **C18.1n9** | 2.08 | 4.09 | 5.24 | 2.81 | 4.8 |
| **C18.1n7** | 2.4 | 2.58 | 2.1 | 1.79 | 2.94 |
| **C18.2n6** | 0.37 | 0.86 | 0.12 | 0.82 | 0.9 |
| **C18.3n3** | 0.19 | 0.38 | 0.94 | 0.28 | 0.52 |
| **C20.1n11** | 1.44 | 2.24 | 1.45 | 1.71 | 1.13 |
| **C20.1n9** | 0.27 | 0.4 | 0.3 | 0.21 | 0.3 |
| **C20.4n6** | 5.78 | 3.09 | 5.22 | 4.39 | 5.05 |
| **C20.5n3** | 18.53 | 18.71 | 16.9 | 17.97 | 16.41 |
| **C22.1n11** | 0.37 | 0.07 | 0.43 | 0.24 | 1.2 |
| **C22.5n3** | 1.49 | 1.48 | 0.9 | 1.77 | 1.2 |
| **C22.6n3** | 25.74 | 28.72 | 31.67 | 29 | 29.05 |
| **C24.1n9** | 0.55 | 0.71 | 0.88 | 0.18 | 1.8 |

**Table S3.** Diet estimations (median and range) produced by MixSIAR for wild squid, based on the original fatty acid data of consumers, or on the simulated data derived from means, standard deviations and sample size of each fatty acid. P values < 0.05 would indicate that estimated diets differ depending on the type of data (original or simulated) used for analysis.

| **Sources** | **Estimated diet proportions** | | ***P* value** |
| --- | --- | --- | --- |
|  | Original data | Simulated data |  |
| Crustaceans | 0.060 (0.003 – 0.312) | 0.059 (0.003 – 0.309) | 0.731 |
| Fish | 0.940 (0.688 – 0.997) | 0.941 (0.691 – 0.997) | 0.731 |
