# Supplementary material for: Evaluating the performance of the Bayesian mixing tool MixSIAR with fatty acid data for quantitative estimation of diet
Source: Sci Rep. 2020 Nov 27;10:20780. doi: 10.1038/s41598-020-77396-1 (PMC7695706; doi:10.1038/s41598-020-77396-1)
Supplement: Supplementary file 3 — Supplementary Information 3. [file 41598_2020_77396_MOESM3_ESM.docx]

**Evaluating the performance of the Bayesian mixing tool MixSIAR with fatty acid data for quantitative estimation of diet**

Alicia I. Guerrero^a^ and Tracey L. Rogers^b^

**SUPPLEMENTARY MATERIAL 3**

^a^ Centro de Investigación y Gestión de Recursos Naturales (CIGREN), Instituto de Biología, Facultad de Ciencias, Universidad de Valparaíso. Gran Bretaña

1111, Playa Ancha, Valparaíso, Chile.

^b^ Evolution and Ecology Research Centre, School of Biological, Earth and Environmental Sciences, University of New South Wales, Sydney, Australia 2052

*Corresponding author:

Alicia I. Guerrero

alicia.guerrero@uv.cl

**Posterior distribution tables**

**Table 1.** MixSIAR predictions of diet composition for spectacled eiders after 69 days on an initial diet consisting of 1% clam, 3% krill, 88% Mazuri sea duck formula, 4% mussel and 4% silverside; 21 days on diet A, consisting of 56% krill and 44% Mazuri formula, and 29 days on diet B, consisting of 48% Mazuri formula and 52% silverside.

| **Actual diet** | **Estimated diet proportions** | | | | |
| --- | --- | --- | --- | --- | --- |
|  | Clam | Krill | Mazuri | Mussel | Silverside |
| Initial diet | 0.001  (0.000–0.020) | 0.042  (0.023–0.066) | 0.885  (0.868–0.899) | 0.063  (0.022–0.074) | 0.010  (0.005–0.018) |
| Diet A | 0.001  (0.000-0.004) | 0.281  (0.254-0.306) | 0.619  (0.600-0.637) | 0.044  (0.032-0.080) | 0.053  (0.037-0.064) |
| Diet B | 0.001  (0.000-0.015) | 0.073  (0.051-0.093) | 0.616  (0.599-0.634) | 0.039  (0.008-0.049) | 0.272  (0.255-0.289) |

**Table 2.** MixSIAR predictions of diet composition for spectacled eiders after 69 days on an initial diet consisting of 88% Mazuri sea duck formula, 1% krill, 3% silverside, 1% clam, and 7% mussel; 21 days on diet A, consisting of 34% Mazuri formula and 66% krill, and 29 days on diet B, consisting of 34% Mazuri formula and 66% silverside.

| **Actual diet** | **Estimated diet proportions** | | | | |
| --- | --- | --- | --- | --- | --- |
|  | Clam | Krill | Mazuri | Mussel | Silverside |
| Initial diet | 0.002  (0.000–0.020) | 0.020  (0.010–0.034) | 0.905  (0.891–0.917) | 0.044  (0.020–0.060) | 0.026  (0.015–0.041) |
| Diet A | 0.002  (0.000-0.024) | 0.456  (0.425-0.496) | 0.274  (0.247-0.395) | 0.219  (0.029-0.253) | 0.056  (0.034-0.041) |
| Diet B | 0.002  (0.000-0.018) | 0.094  (0.073-0.113) | 0.278  (0.250-0.349) | 0.166  (0.087-0.197) | 0.465  (0.437-0.479) |

**Table 3.** Diet estimates for Atlantic salmon fed, for 22 weeks, one of four diets: 100% herring oil, a mixture of 70:30 herring to krill oil, a mixture of 30:70 herring to krill oil or 100% krill oil. Proportions obtained using three sets of CCs: one derived from salmon fed a diet based on krill oil (KO-CC), another where salmon was fed a diet based on herring oil (HO-CC), and a third set of CCs where KO-CC and HO-CC were averaged (Combined-CC).

| **CC** | **Actual diet** | **Estimated diet proportions** | | | |
| --- | --- | --- | --- | --- | --- |
|  |  | Herring oil | | Initial diet | Krill oil |
| Krill oil | Herring oil | 0.808 (0.796 – 0.820) | 0.041 (0.031 – 0.051) | | 0.151 (0.139 – 0.163) |
|  | 70:30 Herring: krill oil | 0.621 (0.611 – 0.631) | 0.044 (0.035 – 0.053) | | 0.335 (0.324 – 0.347) |
|  | 30:70 Herring: krill oil | 0.270 (0.263 – 0.279) | 0.033 (0.023 – 0.042) | | 0.696 (0.684 – 0.709) |
|  | Krill oil | 0.006 (0.002 – 0.011) | 0.019 (0.006 – 0.030) | | 0.975 (0.962 – 0.989) |
| Herring oil | Herring oil | 0.980 (0.970 – 0.991) | 0.001 (0.000 – 0.005) | | 0.018 (0.008 – 0.028) |
|  | 70:30 Herring: krill oil | 0.794 (0.784 – 0.804) | 0.008 (0.002 – 0.015) | | 0.198 (0.188 – 0.208) |
|  | 30:70 Herring: krill oil | 0.409 (0.399 – 0.420) | 0.013 (0.003 – 0.028) | | 0.577 (0.561 – 0.592) |
|  | Krill oil | 0.093 (0.080 – 0.110) | 0.012 (0.001 – 0.037) | | 0.891 (0.868 – 0.912) |
| Combined | Herring oil | 0.895 (0.884 -0.906) | 0.018 (0.009 – 0.027) | | 0.087 (0.074 – 0.099) |
|  | 70:30 Herring: krill oil | 0.709 (0.700 – 0.719) | 0.018 (0.010 – 0.025) | | 0.273 (0.262 – 0.284) |
|  | 30:70 Herring: krill oil | 0.337 (0.328 – 0.345) | 0.010 (0.003 – 0.018) | | 0.653 (0.642 – 0.664) |
|  | Krill oil | 0.047 (0.037 – 0.057) | 0.004 (0.000 – 0.011) | | 0.949 (0.936 – 0.960) |

**Table 6.** Diet estimates for tufted puffin nestlings fed an unknown diet by their parents until day 10, and then herring for the next 27 days.

| **Models** | **Estimated diet proportions** | | | | | |
| --- | --- | --- | --- | --- | --- | --- |
|  | Capelin | Cod | Herring | Salmonid | Sandfish | Sandlance |
| **Day 10, with priors** | 0.150  (0.096 – 0.216) | 0.040  (0.010 – 0.094) | - | 0.027  (0.006 – 0.069) | 0.049  (0.019 – 0.099) | 0.723  (0.640 – 0.798) |
| **Day 10, no priors** | 0.605  (0.033 – 0.696) | 0.255  (0.058 – 0.363) | - | 0.024  (0.001 – 0.142) | 0.026  (0.001 – 0.129) | 0.060  (0.002 – 0.800) |
| **Day 10, no priors, herring** | 0.597  (0.479 – 0.687) | 0.264  (0.138 – 0.367) | 0.009  (0.000 – 0.049) | 0.021  (0.001 – 0.137) | 0.023  (0.001 – 0.114) | 0.053  (0.003 – 0.200) |
| **Day 19, no priors** | 0.322  (0.001 – 0.396) | 0.004  (0.000 – 0.026) | 0.600  (0.556 – 0.652) | 0.002  (0.000 – 0.012) | 0.006  (0.000 – 0.018) | 0.072  (0.008 – 0.378) |
| **Day 28, no priors** | 0.072  (0.000 – 0.157) | 0.001  (0.000 – 0.010) | 0.826  (0.798 – 0.854) | 0.001  (0.000 – 0.007) | 0.003  (0.000 – 0.009) | 0.102  (0.010 – 0.175) |
| **Day 37, no priors** | 0.013  (0.000 – 0.042) | 0.001  (0.000 – 0.006) | 0.973  (0.948 – 0.991) | 0.000  (0.000 – 0.003) | 0.001  (0.000 – 0.006) | 0.008  (0.000 – 0.032) |

**Table 7**. Diet estimations produced by MixSIAR for juvenile harp seals fed Atlantic herring for a year prior to day 0, and then only Atlantic pollock for the successive 30 days. Whereas herring contained ≥9% of fat, pollock only contained 1.7%.

| **Date** | **Estimated diet proportions** | |
| --- | --- | --- |
|  | Herring | Pollock |
| Day 0 | 0.990 (0.978 – 0.998) | 0.010 (0.002 – 0.022) |
| Day 14 | 0.991 (0.971 – 0.998) | 0.009 (0.001 – 0.029) |
| Day 30 | 0.955 (0.935 – 0.989) | 0.045 (0.011 – 0.065) |

**Table 8.** MixSIAR diet estimations for three groups of harbour seals fed different types of diet: Pacific herring for 42 days, surf smelt for 42 days, or smelt for 21 days and then Pacific herring for 21 days. Analyses were performed on FAs from blubber collected at the end of the feeding experiment (day 42). Values are presented as median and range (2.5% and 97.5%)

| **True diet** | **Estimated diet proportions** | | |
| --- | --- | --- | --- |
|  | Herring | Salmon | Smelt |
| Herring (HE) | 0.947 (0.866 – 0.984) | 0.023 (0.001 – 0.101) | 0.025 (0.007 – 0.062) |
| Surf smelt (SM) | 0.726 (0.680 – 0.821) | 0.007 (0.001 – 0.025) | 0.266 (0.166 – 0.313) |
| Smelt – Herring (SMHE) | 0.909 (0.843 – 0.967) | 0.061 (0.001 – 0.126) | 0.030 (0.003 – 0.085) |
